# Hidden Rhythms: A Complex Case of Hyperemesis Gravidarum-Induced Arrhythmias

**DOI:** 10.7759/cureus.75548

**Published:** 2024-12-11

**Authors:** Todd R Anderson, Emily J Carletto, Valeria Barreto-Nadal, Eloise Joubert, David Schutzer

**Affiliations:** 1 Obstetrics and Gynecology, Campbell University School of Osteopathic Medicine, Lillington, USA; 2 Pediatrics, Campbell University School of Osteopathic Medicine, Lillington, USA; 3 Obstetrics and Gynecology, Cape Fear Valley Medical Center, Fayetteville, USA

**Keywords:** cardiac arrythmia, cardiology, hyperemesis gravidarum, nausea and vomiting in pregnancy, obs&gy

## Abstract

Hyperemesis gravidarum (HG) is a severe condition marked by intense nausea and vomiting during pregnancy, which is different from typical morning sickness. It is marked by weight loss exceeding 5% of pre-pregnancy weight, ketonuria, dehydration, electrolyte imbalances, and in some cases, arrhythmias - primarily linked to electrolyte disturbances. Treatment typically involves conservative measures such as small, bland meals, medications like metoclopramide and ondansetron, and correction of electrolyte abnormalities. This case study presents a 29-year-old female, G5P2022, who arrived at the ED with severe nausea, vomiting, intermittent chest pain, and palpitations lasting five days. She was confirmed to be seven weeks and five days pregnant. Her medical history included preeclampsia with severe features and HG in prior pregnancies. Initial evaluations, including CBC, complete metabolic panel, and troponin levels, were unremarkable, with normal electrolytes. However, an ECG revealed multiple arrhythmias. Cardiology and electrophysiology consultations recommended outpatient follow-up. This case highlights the serious risks HG poses to both maternal and fetal health. Although arrhythmias are a recognized complication of HG, this case is notable for their occurrence despite normal electrolyte levels, emphasizing the complex interplay between HG and cardiac function.

## Introduction

Hyperemesis gravidarum (HG) is a severe condition of nausea and vomiting during pregnancy, distinct from typical morning sickness [1]. Diagnostic criteria include a weight loss exceeding 5% of pre-pregnancy weight and ketonuria not attributable to other causes [2,3]. Additional diagnostic criteria are as follows: (1) symptoms begin before 16 weeks of gestation; (2) nausea and vomiting are severe; (3) the patient is unable to eat or drink normally; and (4) daily activities are significantly impaired [4].

Risk factors for HG are similar to those associated with less severe nausea and vomiting during pregnancy. These include a history of migraines, multiple gestations, nulliparity, hydatidiform molar pregnancy, and pregnancies with female fetuses [5,6]. Genetic predisposition also plays a role, with evidence suggesting a link between the genes GDF15 and IGFBP7 and the development of HG. GDF15, expressed in placental trophoblast cells, helps regulate neurons in the hypothalamus and area postrema, while IGFBP7 appears to contribute to placentation and appetite control [7].

In addition to genetic influences, hormonal changes during pregnancy are implicated in HG’s pathogenesis. Elevated estradiol levels and reduced prolactin levels may contribute to its development [8]. Moreover, patients with HG tend to have higher levels of human chorionic gonadotropin compared to those experiencing less severe nausea and vomiting [9].

Initial evaluation of HG should include an assessment of weight, orthostatic blood pressure, heart rate, and confirmation of fetal viability via Doppler or ultrasound. Laboratory evaluations should include serum electrolytes (complete metabolic panel) and urinalysis to check for ketones and specific gravity. Additional tests may include blood urea nitrogen (BUN), creatinine, CBC, liver chemistries, amylase/lipase, phosphorus, magnesium, calcium, and thyroid function tests to determine disease severity.

HG poses significant risks for both maternal and fetal health. Maternal complications include weight loss, dehydration, orthostatic hypotension, electrolyte imbalances, hypovolemia, micronutrient deficiencies, and Wernicke encephalopathy. Fetal complications may include low birth weight, being small for gestational age, preterm delivery, and poor five-minute APGAR scores [10]. Laboratory findings indicative of HG may include hypokalemic hypochloremic metabolic alkalosis, hypomagnesemia, hypocalcemia, ketosis (from minimal caloric intake), increased hematocrit due to volume depletion, elevated BUN, elevated urine specific gravity, elevated alanine aminotransferase and aspartate aminotransferase, elevated serum amylase and lipase, and transient hyperthyroidism.

Management of HG includes nonpharmacologic and pharmacologic interventions. Nonpharmacologic strategies involve consuming small, bland meals, avoiding triggers for vomiting, and using ginger extract [11]. First-line pharmacologic treatments consist of pyridoxine (vitamin B6) alone or in combination with doxylamine. If symptoms persist despite first-line treatment, alternatives include promethazine, dimenhydrinate, diphenhydramine, or prochlorperazine. For patients who are not hypovolemic or dehydrated, metoclopramide, ondansetron, or trimethobenzamide may be considered. In cases of dehydration or hypovolemia, intravenous fluid replacement with isotonic saline or lactated Ringer’s solution is essential [11].

## Case presentation

**Figure 1:**
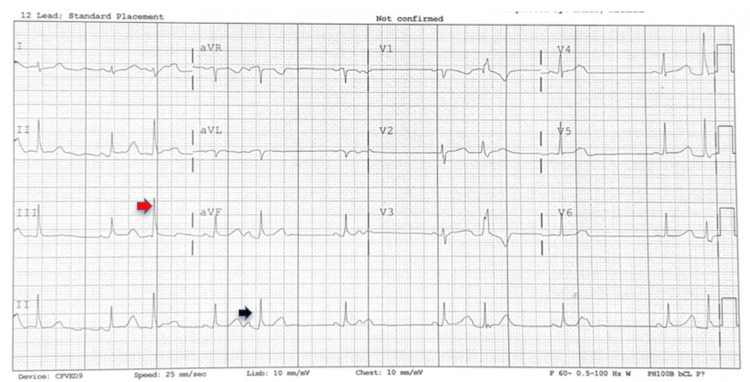
Abnormal ECG showing sinus rhythm with multiple premature ventricular complexes (red arrows) and premature atrial complexes (black arrows)

**Figure 2:**
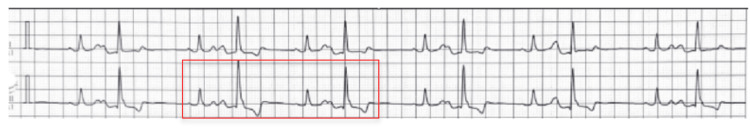
Abnormal ECG showing sinus rhythm with ventricular bigeminy (red box)

**Figure 3:**
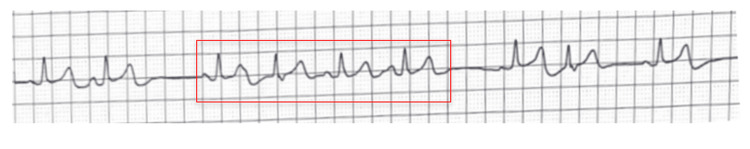
Abnormal ECG showing an irregular rhythm with grouped beating (red box)

This case study presents a 29-year-old female, G5P2022, who presented with severe nausea, vomiting, intermittent chest pain, and palpitations for the past five days. The patient described the chest pain as burning, which worsened with vomiting. She reported the emesis as bile-streaked with blood, without any evidence of coffee-ground material. Her past medical history included preeclampsia with severe blood pressure elevation (Figure 1, Figure 2, Figure 3).

Treatment for HG was initiated with scheduled doxylamine, pyridoxine, promethazine, and as-needed ondansetron. Within a few days of admission, the patient tolerated an oral diet, and her symptoms of nausea and vomiting were controlled. Although the patient continued to experience palpitations, she expressed a desire to be discharged. She was discharged with a cardiology follow-up appointment and a routine prenatal care appointment.

The cardiologist recommended that the patient follow up outpatient after her pregnancy. After delivery, the patient visited the cardiologist, where she was reported to be asymptomatic. A repeated ECG showed normal sinus rhythm with no evidence of arrhythmic activity. The patient and healthcare provider agreed that no further workup was necessary, and the patient was educated on seeking further evaluation if any symptoms reoccurred in the future.

## Discussion

HG can lead to severe complications, including electrolyte imbalances, which may predispose patients to arrhythmias. Prompt recognition and treatment of these imbalances are crucial to prevent adverse cardiac events. Management typically focuses on rehydration, electrolyte correction, and controlling nausea and vomiting to improve maternal and fetal outcomes.

This case is unique because the patient’s electrolytes were within normal limits, whereas arrhythmias in HG are typically associated with electrolyte imbalances. The arrhythmias observed in this case were likely idiopathic, possibly resulting from the stress placed on the heart due to severe nausea and vomiting. Persistent arrhythmias can increase the risk of arrhythmia-induced cardiomyopathy, heart failure, and mortality [12]. Treatment should be initiated after identifying any correctable causes, such as electrolyte abnormalities, substance use, or hypertension. First-line treatments for arrhythmias include beta-blockers [13], while other options include catheter ablation or antiarrhythmics like amiodarone [14,15].

Additionally, this patient had experienced similar issues in previous pregnancies, but the arrhythmias were only present during this pregnancy. After the delivery of her previous pregnancy, the patient underwent a cardiology evaluation, which was negative for arrhythmia. During this pregnancy, another cardiology consult was requested, but due to the patient’s largely asymptomatic status after treatment for nausea and vomiting, further workup was deferred until the postpartum period, as there was hesitancy to conduct additional testing during pregnancy.

This case highlights the challenges in managing arrhythmias during pregnancy. When treating pregnant patients, caution is necessary when selecting medications, taking into account the safety profiles of various drugs. For example, the beta-blocker atenolol is cautioned in pregnancy due to potential adverse effects such as hypoglycemia in the infant and intrauterine fetal growth restriction. Furthermore, cardiac ablation and antiarrhythmics like amiodarone are contraindicated. Therefore, a careful assessment and a multidisciplinary approach are essential to ensure the safest outcomes for both mother and fetus.

## Conclusions

This case of HG complicated by persistent arrhythmias highlights the critical need for thorough cardiac monitoring and management during pregnancy. Despite the patient’s normal electrolyte levels, the presence of arrhythmias suggests that the physical stress of HG alone can trigger significant cardiac concerns, potentially leading to more severe conditions such as arrhythmia-induced cardiomyopathy if left untreated. A multidisciplinary approach, involving collaboration between obstetricians and cardiologists, is essential in managing complex cases like this, where treatment options may be limited by the pregnancy itself.

Additionally, this case underscores the ongoing challenge of balancing effective treatment with the safety of both the mother and fetus. The decision to defer a more aggressive cardiology workup until the postpartum period reflects the cautious approach required when managing pregnant patients, as certain medications and procedures may pose risks. Ultimately, this case emphasizes the importance of individualized care plans that take into account the unique physiological changes of pregnancy, focusing on minimizing risks while addressing potentially life-threatening conditions like arrhythmias in the context of HG.
